# What Large Language Models offer about Familial Mediterranean Fever: An Analysis of Quality, Readability, Completeness, and Accuracy

**DOI:** 10.31138/mjr.261224.hfm

**Published:** 2025-08-20

**Authors:** Burak Tayyip Dede, Didem Erdem Gürsoy, Muhammed Oğuz, Bülent Alyanak, Fatih Bağcıer

**Affiliations:** 1 Department of Physical Medicine and Rehabilitation, Prof. Dr. Cemil Tascioglu City Hospital, Istanbul, Turkey; 2 Department of Rheumatology, Prof. Dr. Cemil Tascioglu City Hospital, Istanbul, Turkey; 3 Department of Physical Medicine and Rehabilitation, Istanbul Training and Research Hospital, Istanbul, Turkey; 4 Department of Physical Medicine and Rehabilitation, Golcuk Necati Celik State Hospital, Kocaeli, Turkey; 5 Department of Physical Medicine and Rehabilitation, Basaksehir Cam and Sakura City Hospital, Istanbul, Turkey

**Keywords:** artificial intelligence, large language model, familial mediterranean fever, health literacy, patient education

## Abstract

**Background:**

The aim of this study was to evaluate the quality, completeness, accuracy, and readability of Large Language Models (LLM) responses to 25 popular questions about Familial Mediterranean Fever (FMF).

**Methods:**

The readability of the responses of LLMs (ChatGPT-4, Copilot, Gemini) was assessed by Flesch Reading Ease Score (FRES) and Flesch-Kincaid Grade (FKG). The Ensuring Quality Information for Patients (EQIP) tool was used to assess the quality. To assess the completeness and accuracy of responses, 3-point and 5-point Likert scales were used, respectively.

**Results:**

The mean FRES scores of LLMs ranged between 29.80 and 35.66. The FKG scores ranged between 12.36 and 13.72. The mean accuracy scores of LLMs ranged between 4.88 and 4.96. No significant difference was found between the LLM groups regarding accuracy and readability scores (p>0.05). The mean completeness scores of LLMs ranged between 2.36 and 2.84. ChatGPT-4 was the leading LLM in completeness scores according to the Likert scale, and the difference between LLM groups was statistically significant (p=0.006). Gemini performed better in the quality analysis with the EQIP tool, and there was a statistically significant difference between the LLM groups (p<0.001).

**Conclusion:**

In this study, LLMs performed acceptably in accuracy and completeness. However, there are serious concerns about their readability and quality. To improve health information, LLM developers should include more diverse data sources in the training sets of the models. Moreover, the ability of LLMs to provide readability features that are adaptable to the level of education could be an important innovation in this field.

## INTRODUCTION

Advances in artificial intelligence have impacted medicine and healthcare over the last few years. As a subclass of artificial intelligence, Large Language Models (LLMs) have significant potential in rheumatology. Their various applications and benefits, including improving diagnostic accuracy and personalising patient care, make them one of the most discussed topics recently.^1–3^ Studies have demonstrated the contributions of LLMs to rheumatologists in clinical practice, research, and medical education.^4^ On the other hand, it can improve the health literacy of patients with chronic rheumatic diseases.^5^ LLMs can potentially contribute to patient education by providing detailed and empathetic answers to their questions and accurate and easy-to-understand information about their conditions and treatments.^1,6,7^

Several studies have been conducted in various fields of rheumatology to evaluate the performance of various LLMs in responding to medical queries. ChatGPT models have shown substantial potential for providing accurate and complete patient information in the context of methotrexate use in rheumatology.^8^ However, the possibility of hallucinatory and misleading information about drugs has also been noted.^6,8^ Another study has demonstrated that GPT-4 was associated with higher quality and empathy in responding to frequently asked questions from patients with systemic lupus erythematosus compared to experts.^9^ On the contrary, it has been demonstrated that ChatGPT’s answers regarding the application of complementary and alternative medicine methods in selected rheumatic diseases were not convincingly based on scientific evidence.^10^ When comparing the performance of LLM chatbots to physician-generated responses to rheumatology questions, it was shown that despite patients rating the responses as similar in quality, rheumatologists found chatbot-generated responses to be inferior to rheumatologist-generated responses.^11^ Considering all this, patients should be careful when using these LLM tools to address their rheumatological medical questions.

Familial Mediterranean fever (FMF) is the most frequent autoinflammatory disease with autosomal recessive inheritance and can result in the development of secondary amyloidosis.^12^ FMF patients and their relatives frequently use online platforms for medical information about the disease, disease course, treatment, and attacks. Today, LLM provides faster and easier access to medical information that can lead to patient education, early diagnosis, management of attacks, and treatment adherence. However, the accuracy and quality of LLM’s answers to medical questions about FMF disease need to be evaluated. To our knowledge, there is no study evaluating the information provided by LLMs for FMF disease. This study aimed to evaluate the quality and performance of three different LLM in responding to questions about FMF disease in terms of accuracy, completeness, quality, and readability.

## METHODS

This study was designed as a cross-sectional observational study. Since the present study does not involve patient records or human specimens, ethics committee approval was not required.

Three different LLMs were evaluated (ChatGPT-4, Co-pilot, Gemini). ChatGPT (Chat-Generative Pre-Trained Transformer) (OpenAI Inc.) was launched in November 2022. Copilot (Bing AI), one of the other popular LLMs, was launched by Microsoft in February 2023, and Gemini (Google Bard) was launched by Google in March 2023.^13^

### Determining the questions and obtaining the LLMs-generated answers

To determine the questions to be asked to LLM programs, the question “Can you provide the 25 most frequently asked questions about familial mediterranean fever according to Google Trends?” was asked in English to the Gemini chatbot of Google Limited Liability Company. In the process of creating the questions, it was thought that the integration of the Gemini, which is launched within the same company as Google, a very popular search engine worldwide, could be better. Then, a clinician analysed the 25 questions provided by Gemini for grammar and spelling.

On 21.08.2024, these 25 different questions were asked by a clinician (BTD) to ChatGPT-4, Copilot, and Gemini tools separately in the same session, and the responses were recorded. Responses were assessed by two clinicians with experience in rheumatic diseases blinded to the LLM from which the responses were collected (BA, MO). When there was irreconcilability in the assessments of the clinicians, the other two clinicians (DEG, FB) were consulted, and a consensus was reached.

### Evaluation of readability

Flesch Reading Ease Score (FRES) and Flesch-Kincaid Grade (FKG) were used to evaluate the readability of the responses. The FRES has a scale of 0–100, with a higher score indicating better readability. FKG presents the US reading level; a lower score indicates a more readable text.^14^ For FRES, a score > 60 has been reported as the appropriate readability level for patient groups; for FKG, a score < 8 has been reported as the appropriate score for universal accessibility and readability.^15^

### Evaluation of quality

The Ensuring Quality Information for Patients (EQIP) tool was used to assess the quality of responses. The EQIP tool is a reliable tool that consists of 20 questions and assesses the quality of written information in the health field.^16^ The evaluator answers the questions in a way to express 1; Yes, 0.5; Partly, 0; No. Unanswered questions are not evaluated. The scores are summed according to the questions answered, divided by the number of questions answered, and multiplied by 100 to calculate the EQIP score. The EQIP categorises scores between 76% and 100% as ‘well written’ and excellent quality, scores between 51% and 75% as ‘good quality with minor problems’, scores between 26% and 50% as serious quality problems’, and scores between 0% and 25% as severe quality problems’.^17^

### Evaluation of completeness

A 3-point Likert scale was used to assess the completeness of the responses. 1 indicates incomplete information, 2 indicates adequate information, and 3 indicates comprehensive information.^18^

### Evaluation of accuracy

A 5-point Likert scale was used to evaluate the accuracy of the answers. The scale was delineated as follows: 1 is very inaccurate, 2 is inaccurate, 3 is neither accurate nor inaccurate, 4 is accurate, and 5 is very accurate.^19,20^

### Statistical Analysis

Statistical analysis was performed using the IBM SPSS version 22.0 software (IBM Corp., Armonk, IL, USA). Normal distribution was evaluated with kurtosis-skewness values and the Kolmogorov-Smirnov/Shapiro-Wilk test. Mean, standard deviation and median (min-max) were used for descriptive analyses. ANOVA or the Kruskal-Wallis test was used to compare the differences between the groups. Post-hoc tests were used to make pairwise comparisons between each LMM when significant differences were obtained. Tukey HSD and Dunn’s test was used for post-hoc analysis. P-values below 0.05 were considered as statistically significant results.

## RESULTS

This study evaluated the LLMs’ responses to 25 FMF-related questions. **Table 1** presents the 25 questions. According to the FRES scores of the LLMs’ answers to the questions, ChatGPT-4: mean 29.80 (SD:10.20), Copilot: mean 35.66 (SD:11.00), and Gemini: mean 34.12 (SD:12.97). No statistically significant difference was found between the groups (p > 0.05). The FKG scores of the LLMs’ responses were ChatGPT-4; mean 13.72 (SD:1.96), Copilot; mean 12.36 (SD:1.97), and Gemini; mean 12.74 (SD:2.42). No statistically significant difference was found between the groups regarding the scores of FKG (p > 0.05).

**Table 1.** 25 popular questions about FMF posed to LLMs.

**Rank**	**Question**	**Category of the Topic Based on EQIP**
1	What is Familial Mediterranean Fever (FMF)?	Condition or Illness
2	What are the symptoms of FMF?	Condition or Illness
3	How is FMF diagnosed?	Test Operation Investigation or Procedure
4	What causes Familial Mediterranean Fever?	Condition or Illness
5	Is FMF hereditary?	Condition or Illness
6	How is FMF treated?	Medication or Product
7	What are the medications for FMF?	Medication or Product
8	Can FMF be cured?	Prevention or After Care
9	How can I manage FMF attacks?	Prevention or After Care
10	What is the FMF diet?	Miscellaneous
11	How does FMF affect daily life?	Miscellaneous
12	Can people with FMF live a normal life?	Miscellaneous
13	What is the life expectancy of someone with FMF?	Condition or Illness
14	Can FMF cause complications?	Condition or Illness
15	Is there a cure for FMF?	Prevention or After Care
16	What is the difference between FMF and gout?	Condition or Illness
17	Can FMF be triggered by stress?	Miscellaneous
18	Can FMF cause infertility?	Condition or Illness
19	Is FMF contagious?	Condition or Illness
20	What is the prognosis for FMF?	Condition or Illness
21	What is the FMF gene?	Condition or Illness
22	Can children get FMF?	Condition or Illness
23	Is FMF more common in certain ethnic groups?	Condition or Illness
24	How is FMF related to inflammation?	Condition or Illness
25	Can FMF be prevented?	Prevention or After Care

**Figure 1.**
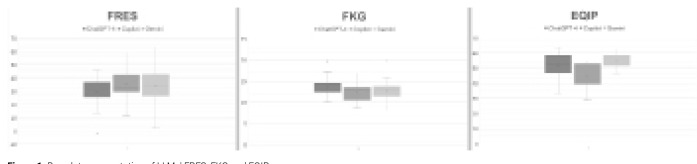
Box plot representation of LLMs' FRES, FKG and EQIP scores.

As a result of the quality assessment with the EQIP tool, Gemini (Median: 54.54, Min: 46.42, Max: 62.50) was the LLM with the best EQIP score (**Figure 1**). According to the EKIQ score, Gemini was followed by ChatGPT-4 (Median: 53.12, Min: 33.33, Max: 63.33) and Copilot (Median: 42.85, Min: 28.57, Max: 54.54). EQIP tool scores were significantly different between the groups (p<0.001). In the analysis between the groups, a statistically significant difference was found between ChatGPT-4 and Copilot (p = 0.006) and between Gemini and Copilot (p < 0.001). However, the difference between ChatGPT-4 and Gemini was not statistically significant (p > 0.05).

**Figure 2.**
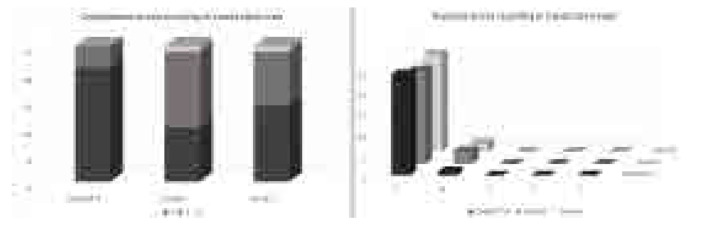
Distribution of the answers given to the questions on a 3-point Likert scale according to the degree of completeness. and distribution of the answers given to the questions on a 5-point Likert scale according to the degree of accuracy.

ChatGPT-4 answered 88%, Copilot 44%, and Gemini 60% of the questions with full proficiency (3 points). The mean completeness scores of the responses obtained from ChatGPT-4, Gemini and Copilot were 2.84 ± 0.37, 2.52 ± 0.59 and 2.36 ± 0.57, respectively (**Figure 2**). Completeness scores assessed using the 3-point Likert scale showed a significant difference between the groups (p = 0.006). In pairwise comparisons, a statistically significant difference was found between ChatGPT-4 and Copilot (p = 0.003). There was no significant difference between the other groups regarding completeness scores (p > 0.05) (**Table 2**).

**Table 2.** Comparison of FRES, FKG, EQIP scores of LLMs and their completeness and accuracy scores according to Likert scale.

	**ChatGPT-4**	**Copilot**	**Gemini**	**p**
**FRES**				
(Mean ± SD)	29.80 ± 10.20	35.66 ± 11.00	34.12 ± 12.97	0.180^a^
Median (Min-Max)	29.40 (−1.70–45.90)	33.10 (11.10–59.30)	33.50 (2.00–63.80)	
**FKG**				
(Mean ± SD)	13.72 ± 1.96	12.36 ± 1.97	12.74 ± 2.42	0.074^a^
Median (Mix–Max)	13.30 (10.00–19.70)	12.80 (8.60–16.90)	13.10 (8.20–19.80)	
**EQIP**				
(Mean ± SD)	51.75 ± 8.15*	44.34 ± 7.99**^**	54.77 ± 4.65	**<0.001^k^**
Median (Min–Max)	53.12 (33.33–63.33)	42.85 (28.57–54.54)	54.54 (46.42–62.50)	
**Completeness**				
(Mean ± SD)	2.84 ± 0.37*	2.36 ± 0.57	2.52 ± 0.59	**0.006^k^**
Median (Min–Max)	3.00 (2.00–3.00)	2.00 (1.00–3.00)	3.00 (1.00–3.00)	
**Accuracy**				
(Mean ± SD)	4.96 ± 0.20	4.88 ± 0.33	4.92 ± 0.28	0.585^k^
Median (Min–Max)	5.00 (4.00–5.00)	5.00 (4.00–5.00)	5.00 (4.00–5.00)	

LLM: Large Language Models; FRES: Flesch-Kincaid Reading Ease Score; FKG: Flesch-Kincaid Grade Level; EQIP: Ensuring Quality Information for Patients; a: ANOVA; k: Kruskal-Wallis test.

* Significant difference between ChatGPT-4 and Copilot, p<0.05

^ Significant difference between Copilot and Gemini, p<0.001

ChatGPT-4 answered 96%, Copilot 88%, and Gemini 92% of the questions completely correctly. The mean accuracy scores of ChatGPT-4, Copilot, and Gemini assessed with the 5-point Likert scale were between 4.88 and 4.96. No statistically significant difference was found between the groups in terms of accuracy scores (p > 0.05) (**Table 2**).

## DISCUSSION

This study, which evaluated the three different LLMs-generated responses to frequently asked questions about FMF, revealed that the LLMs had low readability scores, completion scores with potential for improvement, accuracy scores with potential for excellence, and severe quality issues, especially for Copilot.

It is thought that the prevalence of online health searches in European Union countries has approximately doubled in the last 10 years.^21^ In -light of recent revolutionary developments in the field of GenAI, GenAI technologies such as ChatGPT-4, Gemini, and Copilot are thought to play an important role in the near future to make healthcare services more accessible and efficient. However, there are still significant doubts about ethical concerns, data privacy, and security.^22^ On the other hand, the readability, quality, and reliability of the data available on online platforms, which can be considered partially unregulated, should be evaluated.

In this study, ChatGPT-4, Copilot, and Gemini showed poor readability when their responses to questions on FMF were evaluated with FKG and FRES scores. The score of > 60 for FRES and <8–10 for FKG has been determined as a target score for good readability.^15^ However, the mean FRES and FKG scores in our study were far from these target values. In a previous study on the use of platelet-rich plasma therapy in the treatment of osteoarthritis, ChatGPT-4 was reported to have low readability according to FRES and FKG scores.^23^ In another study evaluating the readability of patient education materials on cardiac catheterisation, ChatGPT, Copilot, Gemini, and Meta AI were assessed with FRES and FKG scores. According to this study, all 4 LLMs failed to reach the target FRES and FKG scores regarding readability.^24^ Our data was also consistent with the literature. Considering the fact that LLMs are generally difficult to read and are open to the use of people from all educational levels, the current situation can be interpreted as worrying. It may be useful for LLM developers to update LLMs to produce more readable texts or algorithms that give variable outputs in terms of readability according to the educational level of the interlocutor.

The present study assessed the quality of responses with the EQIP tool. According to the evaluation made with the EQIP tool, Gemini and ChatGPT-4 were in the good quality category with minor problems, while Copilot was in the serious quality issues category. In Durmaz Engin et al.^21^ study on the role of LLMs in family education about Retinopathy of Prematurity, the EQIP tool scores of LLMs varied between 61.1 and 72.2, and LLMs performed better than in our study. Another study evaluating ChatGPT responses about osteoporosis reported a result close to our study with a mean EQIP score of 48.71.^25^ This discrepancy in the literature may be due to the fact that GenAI-based LLMs are structured to process new data inputs and change their outputs over time. In addition, LLMs may contain different quality data loads for different diseases in existing databases that have the potential to produce outputs. As a result, as supported by the existing literature, it may be better to be sceptical about the quality of the answers provided by LLMs in the field of health for now.

Completeness has always been the most frequently used and essential quality assessment factor.^26^ According to the completeness scores in this study, ChatGPT-4 was generally more successful than Copilot and Gemini. In a recent study that evaluated LLMs in answering questions about methotrexate in the treatment of rheumatoid arthritis, ChatGPT-4 demonstrated superiority over other language models in terms of completeness, which is also the case in our study.^8^

Additionally, a mean completeness score of 2.5 was reported in a study in which 284 medical questions from 17 specialties were directed to ChatGPT.^18^ In another study evaluating ChatGPT-generated information on interceptive orthodontics, the completeness score of open-ended questions directed to ChatGPT was reported as 2.4 out of 3.^27^ ChatGPT-4 performed slightly better in the questions related to FMF than in studies on other medical conditions in the literature. However, considering that the information provided in the health field should be extremely comprehensive and complete, it can be considered that all LLMs, Copilot in particular, need improvement in completeness.

In the accuracy evaluation of the answers, ChatGPT-4 answered 96% of the questions, Copilot answered 88%, and Gemini answered 92% of the questions with full accuracy and provided answers with high accuracy rates. Although there was no statistical difference in the accuracy scores of LLMs in our study, there were differences between the accuracy scores of LLMs in favour of ChatGPT-4 in the study of Durmaz Engin et al..^21^ In this study, which evaluated the responses of LLMs to questions about retinopathy of prematurity, ChatGPT-4 answered 90% of the questions, Copilot answered 30% of the questions, and Gemini answered 38% of the questions correctly at the “strongly agree” level. The correct answer was at the “strongly agree” level. On the other hand, according to a study investigating the diagnostic accuracy of ChatGPT-4 in rheumatology, the researchers emphasised that ChatGPT-4 may have a digital triage potential.^28^ The accuracy scores in our study supported this potential of ChatGPT-4. However, considering the readability, quality, and adequacy scores, it can be considered that this potential should be improved. Following the launch of a website providing high-quality online health information by the Dutch College of General Practitioners in March 2012, it was reported that health service utilisation in the Netherlands decreased by 12% over a two-year period.^29^ With LLMs reaching a level where they can provide high-quality, readable, and accurate information, reducing healthcare costs and providing more effective healthcare services may be possible.

The diagnostic usefulness and competence of LLMs have been frequently discussed in the literature. Although some promising results have been reported, there are still some question marks due to hallucination potential, algorithmic bias, and the need for clinical validation.^30^ In a study evaluating the performance of LLMs in the differential diagnosis of FMF and IL-1 receptor antagonist deficiency, LLMs performed successfully, although not as well as an experienced physician.^31^ Although the diagnostic competence of LLMs was not directly evaluated in this study, Gemini and ChatGPT-4 received 5 points, and Copilot received 4 points on the 5-point Likert scale in the answers to the question “How is FMF diagnosed?”. On the proficiency scale, all LLMs scored 2 points on the 3-point Likert scale. As a result, it can be concluded that LLMs still need to improve in the information provided about the diagnosis as well as their diagnostic competence in FMF.

Our study showed that ChatGPT-4, Copilot, and Gemini have low readability, with Copilot, in particular, having severe quality issues in responding to FMF-related questions. Data from this study suggests that Copilot, Gemini, and ChatGPT-4 may struggle with completeness and could potentially improve their accuracy. In light of these data, the aim should be for LLMs to produce readable, high-quality, accurate, and complete answers. In this study, questions were posed to LLMs only once in a single session, but it has been reported that ChatGPT-4 can produce responses of different quality depending on the quality of the prompts used; it can even produce different responses when the same prompt is posed by different users.^32^ Therefore, in addition to readable, high-quality, accurate, and complete responses, another goal may be to provide standardised responses on medical topics by LLMs. Patient and family education is critical to manage complications and optimise outcomes in chronic rheumatic diseases such as FMF. Considering that FMF starts in childhood, genetic predisposition, and requires lifelong treatment according to current treatment protocols, patients and families may need additional information at any time.^33^ Making LLMs present readable, high-quality, accurate, adequate, and standardised health responses can significantly contribute to public health by fulfilling the need for an accessible, safe, and inexpensive platform at any time.

This study had some limitations. Firstly, the questions posed to the LLMs and the answers of the LLMs were analysed in one language (English). This creates limitations in how LLMs perform in multicultural and multilingual communities. In addition, for diseases such as FMF where regional prevalence may vary, queries in Turkish, Hebrew, Arabic, Greek, and Armenian may change the results. Future studies should include the assessment of responses in different languages. Moreover, although FMF is a common autoinflammatory disease, its prevalence varies regionally. This may have reduced the representativeness of FMF in the datasets used by LLMs. Accuracy and completeness scores may have been affected by this. On the other hand, the number of questions was limited to 25. Another limitation was that the potential answers of LLMs to other questions less frequently asked by families and patients were not included in the study. The possibility that LLM databases provide more consistent, high-quality, and accurate answers to questions frequently asked by human users should not be ignored. In conclusion, LLMs’ responses to questions about FMF were generally of moderate quality, with acceptable levels of adequacy and accuracy and low readability. Our results are promising for the future. The natural evolution of LLMs to provide high-quality, readable, complete, and accurate information can contribute to public health and treatment costs. However, for now, the routine use of LLMs without expert opinion in a field as important as health carries risks. In this process, rheumatologists should familiarise themselves with the risks and convenience of LLM use, which has become widespread among patients. Until the optimisation of LLMs that offer a digital triage potential is achieved, they should also maybe be positioned as a chef responsible for the confirmation of the information provided to patients by LLMs. This study may encourage the development of a new language model that can produce high-quality content with minimal errors in the health field with the support of international committees of LLM developers.

## CONFLICT OF INTEREST

Burak Tayyip Dede, Didem Erdem Gürsoy, Muhammed Oğuz, Bülent Alyanak, and Fatih Bağcıer declare that they have no conflict of interest.

## FUNDING STATEMENT

None.

## ETHICS APPROVAL AND CONSENT TO PARTICIPATE

Ethics committee approval was not necessary.

## AVAILABILITY OF DATA AND MATERIALS

The authors confirm that data supporting the findings of this paper are available upon appropriate request.

## AUTHOR CONTRIBUTIONS

Concept: BTD, DEG, FB; Design: BTD, MO, BA; Control/Supervision: FB, DEG; Analysis and/or Interpretation: BTD, MO, BA; Data Collection and/or Processing: BTD, DEG, MO, BA, FB; Literature Review: BTD, DEG, BA; Writing the Article: BTD, DEG, MO; Critical Review: DEG, BA, FB. All authors contributed to the study conception and design. All authors read and approved the final manuscript. All authors take full responsibility for the integrity and accuracy of the work.

## DISCLAIMER AND DISCLOSURE OF USE OF ARTIFICIAL INTELLIGENCE

No part of this article, including the text and graphics, is copied or published elsewhere in whole or in part. No artificial intelligence was used for writing or editing the article. However, due to the methodological nature of the study, AI-based Large Language Models were used in the data collection phase of the study. This is detailed in the methods section.
